# Endothelial responses of the alveolar barrier in vitro in a dose-controlled exposure to diesel exhaust particulate matter

**DOI:** 10.1186/s12989-017-0186-4

**Published:** 2017-03-06

**Authors:** Sebastian G. Klein, Sébastien Cambier, Jennifer Hennen, Sylvain Legay, Tommaso Serchi, Inge Nelissen, Aline Chary, Elisa Moschini, Andreas Krein, Brunhilde Blömeke, Arno C. Gutleb

**Affiliations:** 1 Luxembourg Institute of Science and Technology (LIST), Environmental Research and Innovation (ERIN) Department, 41, rue du Brill, L-4422 Belvaux, Grand Duchy of Luxembourg; 2 Department of Environmental Toxicology, University Trier, Universitätsring 15, 54296 Trier, Germany; 3 VITO NV, Environmental Risk and Health Unit, Boeretang 200, 2400 Mol, Belgium

**Keywords:** Air-liquid-interface, in vitro model, Coculture, Diesel exhaust particles

## Abstract

**Background:**

During the last 250 years, the level of exposure to combustion-derived particles raised dramatically in western countries, leading to increased particle loads in the ambient air. Among the environmental particles, diesel exhaust particulate matter (DEPM) plays a special role because of its omnipresence and reported effects on human health. During recent years, a possible link between air pollution and the progression of atherosclerosis is recognized. A central effect of DEPM is their impact on the endothelium, especially of the alveolar barrier. In the present study, a complex 3D tetraculture model of the alveolar barrier was used in a dose-controlled exposure scenario with realistic doses of DEPM to study the response of endothelial cells.

**Results:**

Tetracultures were exposed to different doses of DEPM (SRM2975) at the air-liquid-interface. DEPM exposure did not lead to the mRNA expression of relevant markers for endothelial inflammation such as *ICAM-1* or *E-selectin*. In addition, we observed neither a significant change in the expression levels of the genes relevant for antioxidant defense, such as *HMOX1* or *SOD1*, nor the release of pro-inflammatory second messengers, such as IL-6 or IL-8*.* However, DEPM exposure led to strong nuclear translocation of the transcription factor Nrf2 and significantly altered expression of *CYP1A1* mRNA in the endothelial cells of the tetraculture.

**Conclusion:**

In the present study, we demonstrated the use of a complex 3D tetraculture system together with a state-of-the-art aerosol exposure equipment to study the effects of in vivo relevant doses of DEPM on endothelial cells in vitro. To the best of our knowledge, this study is the first that focuses on indirect effects of DEPM on endothelial cells of the alveolar barrier in vitro. Exposure to DEPM led to significant activation and nuclear translocation of the transcription factor Nrf2 in endothelial cells. The considerably low doses of DEPM had a low but measurable effect, which is in line with recent data from in vivo studies.

**Electronic supplementary material:**

The online version of this article (doi:10.1186/s12989-017-0186-4) contains supplementary material, which is available to authorized users.

## Background

During the last 250 years, the level of exposure to combustion-derived particles increased dramatically in western countries due to industrialization and globalization processes such as increased traffic and shipping activities. This resulted in the presence of considerable quantities of coarse (aerodynamic diameter <10 μm), fine (aerodynamic diameter <2.5 μm) and ultrafine (aerodynamic diameter <0.1 μm) particles in the near surface atmosphere [1].

Among the different types of particles present in the ambient air, diesel exhaust particulate matter (DEPM) plays a special role since it is almost omnipresent in the environment [2]. Diesel engines without particle filters emit up to 100 times more particles compared to gasoline engines equipped with modern exhaust treatment systems [3–6] and in Europe diesel engines power 41% of all cars [7]. The particles produced by diesel engines are often between 10 and 30 nm but they can also agglomerate, and depending on the type of engine particles between 1 and 2 μm have been reported [8]. The outer shell of these particles is known to contain metals, such as copper or zinc and a multitude of polycyclic aromatic hydrocarbons (PAHs), such as anthracene or benzo[a]pyrene (B[a]P) (reviewed in [8]).

Due to their size DEPM are able to reach the alveolar region [9]. As a consequence of the low local metabolism of alveolar cells [10] the adsorbed organic chemicals may translocate into the circulation [11, 12] and affect the endothelial cells of the blood vessels. During recent years, the link between air pollution and the progression of atherosclerosis was elucidated [13, 14]. Interestingly, the number of hospital admissions for pulmonary diseases and cardiovascular injury correlates well with PM levels in ambient air, especially during periods with high levels of particles (reviewed in [15]).

Endothelial dysfunction plays a central role in the pathogenesis of several cardiovascular diseases [16] and it precedes, in general, cardiovascular disease [17]. The mechanisms involved in the process of cardiovascular injury due to exposure to PM still need further evaluation, but there is accumulating evidence that exposure to air pollution affects vascular homeostasis and contributes to the progression of vascular systemic diseases within considerably short time [13]. In the lung, the capillary endothelium is in close contact with the alveolar epithelium [18] and endothelial cells need to be considered as secondary or indirect target. It is commonly accepted that tissue damage due to increased oxidative stress is one of the principal mechanisms of PM-induced sub-chronic pulmonary inflammation (reviewed in [19]. The observed reactive oxygen species (ROS) may be a result of the adsorbed metals, such as chromium, copper or zinc, which may catalyze the formation of ROS in cellular systems (reviewed in [20]). Another possibility to explain the oxidative potential of DEPM is to consider the potential of the adsorbed PAHs to act as ligands of the aryl hydrocarbon receptor (AhR), activating *CYP1A1* gene expression [21] and leading to intracellular ROS formation [22].

**Fig. 1:**
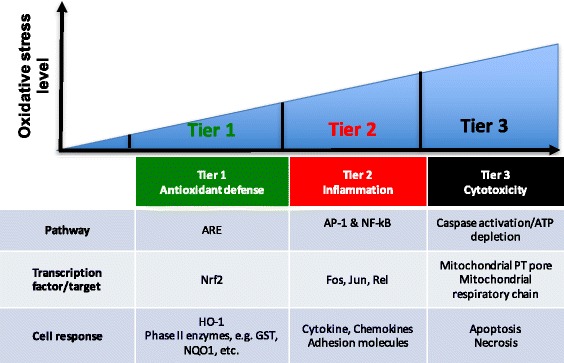
The concept of the hierarchical oxidative stress response. In order to explain the observed effects in cells after exposure to DEPM, the concept of the hierarchical oxidative stress response was developed [23]. In this concept, the cellular response is divided in three different tiers, depending on the level of oxidative stress. In *tier 1* the level of oxidative stress is moderate and cellular defense mechanisms are able to cope with the radicals. In *tier 2* the level of oxidative stress causes already an inflammatory response and activation of cells. Finally, in *tier 3* the oxidative stress leads to cytotoxicity and cellular damage (adapted and modified from [73])

In order to explain the adverse effects of particulate air pollution by DEPM, the concept of the hierarchical oxidative stress response was developed [23]. In brief, this concept can be seen as a tiered response, where cells react differently in respect to the amount of oxidative stress. In *tier 1*, a moderate level of oxidative stress triggers the antioxidant defense, orchestrated by the transcription factor Nrf2. In *tier 2*, oxidative stress leads to the elicitation of inflammation characterized by the expression of surface adhesion molecules and second messengers. In *tier 3*, oxidative stress overcomes cellular defense capacities and leads to extensive cell damage and necrosis [24] (Fig. 1).

Up to now most of the studies with DEPM focused on evaluating the effects of DEPM on relevant target cells using direct exposure scenarios with particle suspensions (reviewed in [25]). For the in vitro analysis of the hazardous potential of DEPM and mechanisms involved it stands to reason to combine the primary targets of DEPM, such as pulmonary epithelial cells, macrophages [26, 27] and mast cells [28, 29], together with endothelial cells, in a 3D coculture system. The possibility of interaction between the different target cell types for DEPM may change the observed effects and thereby result in a more realistic model and better evaluation of the hazardous potential of DEPM on endothelial cells. We here report on the set up of such a model fulfilling the requirements for both, direct exposure of epithelial cells and indirect exposure of endothelial cells [30]. In brief, the model (comprising human cell lines) is composed of A549 epithelial cells, which share many characteristics with alveolar type II cells such as surfactant production and cytokine production, EA.hy 926 endothelial cells, HMC-1 mast cells and THP-1 macrophages [30, 31].

In the present study, this 3D tetraculture model was used in a dose-controlled exposure scenario with realistic doses of DEPM to study the response of endothelial cells after indirect exposure. Doses of 40 and 80 ng/cm^2^ were chosen as both particles doses could be aspirated by a normal adult person during a single day in polluted areas and 240 ng/cm^2^ as a more extreme scenario including extensive physical activity outdoors [32, 33]. Afterwards, endpoints relevant to the concept of the hierarchical oxidative stress response [23] which was proposed to be applicable to the alveolar endothelium [34] were evaluated in these cells.

## Results

### Aerosol characterization – particle counting

**Fig. 2:**
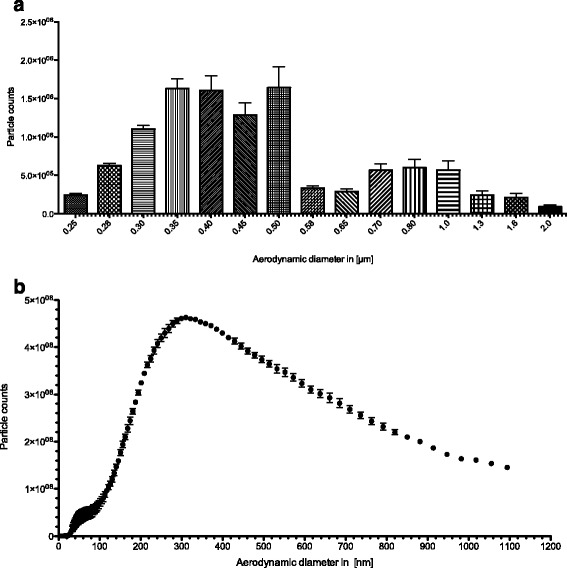
Size distribution of the DEPM aerosol that was produced with the PALAS powder aerosol generator and analyzed using particle counters (optical GRIMM Aerosol counter and SMPS + C counter by GRIMM Aerosol). Particle counts were registered for one minute with the rotating steel brush at 1200 rpm and the piston at 30 mm/h. Data represents the mean of three independent experiments ± SEM. **a**) measurement of the nano-range from 250 nm to 2 μm performed on an optical particle counter by GRIMM Aerosol **b**) measurement of the nano-range from 5 nm to 300 nm performed on a Sequential Mobility Particle Sizer and Counter (SMPS + C) by GRIMM Aerosol

The production of combustion-derived aerosols involves processes, such as nucleation and condensation of the produced particles, which results in the formation of a highly heterogeneous particle-size distribution [35]. In order to verify if the DEPM obtained from the National Institute of Standards and Technology (NIST, Gaithersburg, USA) was suitable to produce a dry-powder aerosol with particles of a relevant size range when aerosolized with the PALAS steel-brush-generator, the aerosol was analyzed by an optical particle counter (GRIMM Aerosol laser spectrometer). Applied DEPM aerosols were evaluated for their particle size distribution and number of particles. The size distribution of the DEPM SRM2975 aerosol was found to be quite heterogeneous (Fig. 2). The main fraction was around 450-500 nm and the upper size limit of the aerosol particles was around 2 μm, which is in line with the cut-off level defined for the cyclone (particle fractionator) in the used PALAS steel-brush-generator. With having the majority of particles below 500 nm in aerodynamic diameter, the produced aerosol is in a relevant range where particles could penetrate deep into the lungs [9].

**Fig. 3:**
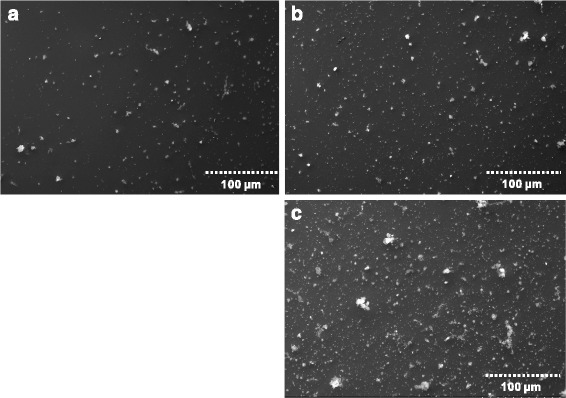
Scanning electron microscopy micrographs of empty Transwell™ inserts after exposure to DEPM aerosol using the PALAS steel-brush-generator. Transwell™ inserts were pre-wetted and then exposed to: **a**: 40 ng/cm^2^; **b**: 80 ng/cm^2^; **c**: 240 ng/cm^2^

In order to assure a uniform exposure of the cells at the air-liquid-interface (ALI), empty Transwell™ inserts were exposed to DEPM under the same conditions as used for the tetracultures (see materials and methods) and the spatial distribution of particles impacting on the membranes was analyzed by scanning electron microscopy (SEM). SEM results confirmed the heterogeneous size distribution with some bigger particles (Fig. 3) and a large proportion of small particles. With increased exposure time, also the likelihood to deposit larger particles was increased. Overall, the distribution of DEPM was uniform for all evaluated doses.

### Presence of metals in DEPM analyzed by NanoSIMS

DEPM are known to carry metals in their outer shell. These metals, such as nickel, copper, chromium or zinc, are supposed to be in particular responsible for the pro-oxidative potential of DEPM [20]. However, the composition and presence of metals is influenced by a multitude of different factors such as fuel, additives and engine type (reviewed in [8]). Regarding the SRM2975, NIST provides a reference material well-characterized for size distribution and PAHs content, but data about the content of metals are missing. In this experiment the presence of metals on the outer shell of DEPM was analyzed by a secondary ion mass spectrometer (NanoSIMS50). This spectrometer offers the possibility to visualize the metal content of the DEPM that were collected on empty Transwell inserts as described above.

**Fig. 4:**
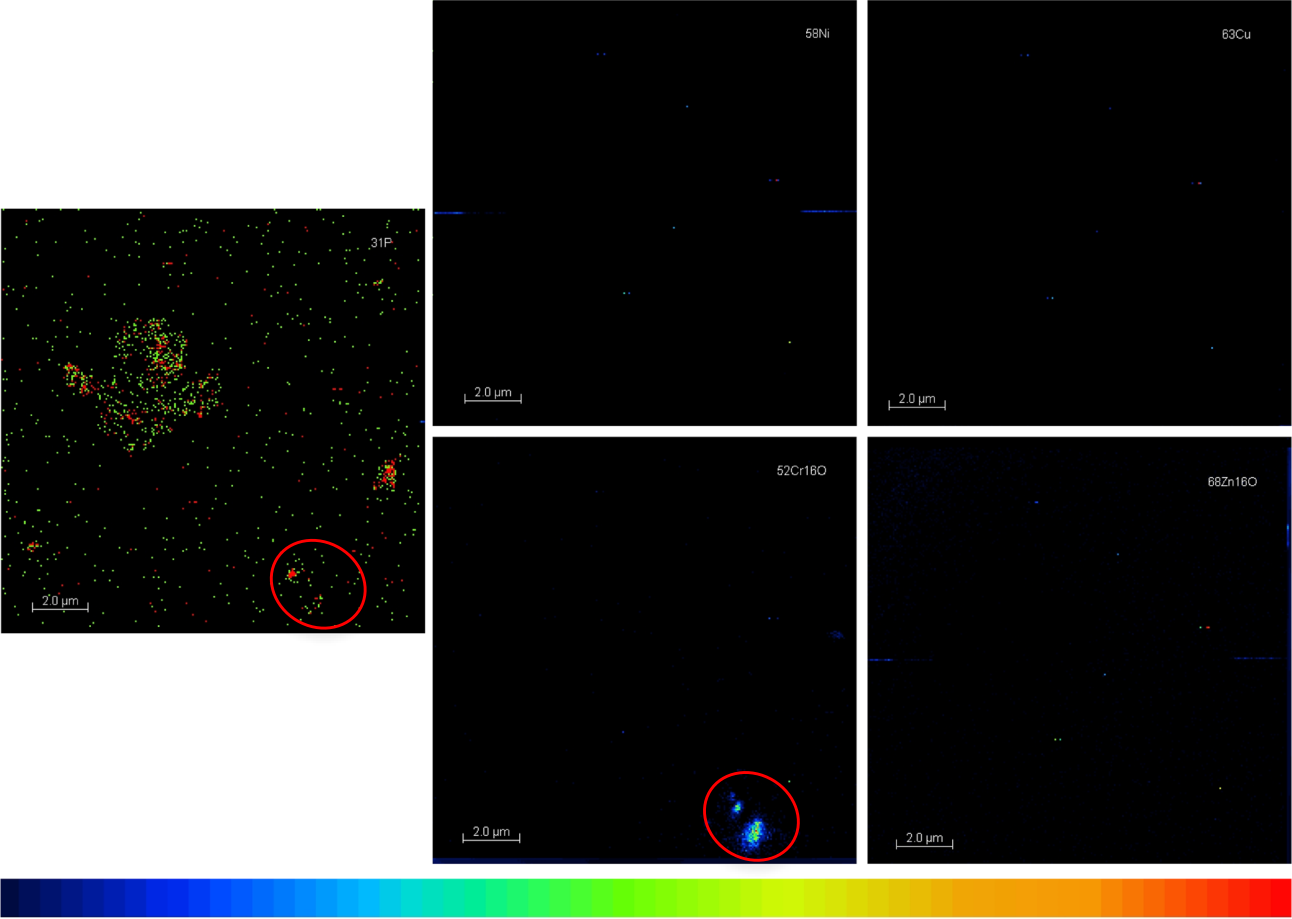
Distribution of metals on the outer shell of DEPM analyzed by NanoSIMS50. DEPM samples were analyzed for the presence of Ni, Cu, Cr and Zn. P was used as reference to draft an image of the outer shell of the particle. The relative amounts of the elements are presented as intensities with a logarithmic scale from black (lowest) to red (highest)

Representative transwell inserts exposed to DEPM were analyzed for the presence of the nickel (Ni), copper (Cu), chromium (Cr) and zinc (Zn) (Fig. 4). Low quantities of chromium were only found in a fraction of particles (see red circle in Fig. 4), whereas the amounts of the evaluated metals was under the limit of detection in the majority of SRM2975 DEPM.

### Influence of DEPM exposure on cellular viability of the tetraculture

**Fig. 5:**
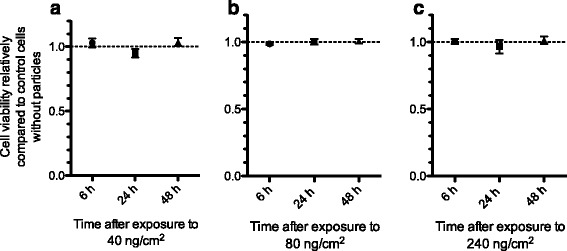
Impact of the exposure to different amounts of DEPM on the cellular viability of the tetraculture at different time-points after exposure. Tetracultures were exposed to 40 ng/cm^2^ (**a**), 80 ng/cm^2^ (**b**) or 240 ng/cm^2^ (**c**) of DEPM and the viability of the tetracultures was evaluated at 6, 24 and 48 h after exposure. DEPM were not significantly affecting the viability of the tetracultures compared to the control cells at any of the evaluated time-points. Tetracultures that without particles served as control (dotted line). Data represents the mean of three independent experiments ± SEM

The viability of the tetracultures was evaluated at 6, 24 and 48 h after exposure by measuring the conversion of resazurin into resorufin, giving an overview on the combined viability of all cell types in the tetraculture. Results are expressed as relative viability compared to the control cells without particles. DEPM were not significantly affecting the viability of the tetracultures compared to the control cells at any of the evaluated time-points (Fig. 5).

### Influence of DEPM exposure on general stress response of the tetraculture

The endothelial cells of the tetraculture showed a significant increase in the expression of *HSP70* mRNA at 24 and 48 h after the indirect exposure to 80 ng/cm^2^ of DEPM (1.36 ± 0.05; 1.27 ± 0.09 fold), compared to the cells analyzed at 6 h after the exposure, which were at the level of the control cells (0.99 ± 0.11 fold) (P < 0.05) (Table 1). Cells treated with 240 ng/cm^2^ showed the highest response for the upregulation of *HSP70* mRNA with 1.51 ± 0.06 fold increase (*P* < 0.05 compared to untreated control cells) after 48 h (Table 1).

**Table 1:**
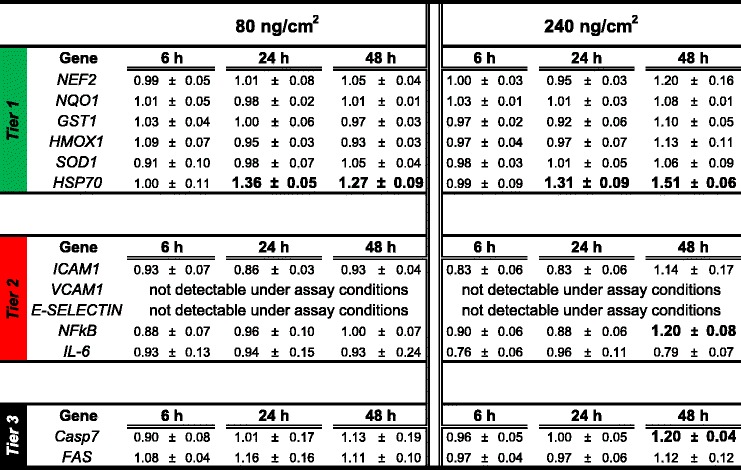
Differential gene expression of relevant markers of the oxidative stress tier approach in the endothelial cells of the tetraculture 6, 24 and 48 h after exposure to DEPM (80 ng/cm^2^ or 240 ng/cm^2^)

Results were normalized to the level of expression of the control cells without particles. Data represent the mean of at least four independent experiments ± SEM. Bold letters indicate significant differences compared to untreated controls (*P*<0.05)

### Hierarchical oxidative stress response in endothelial cells

#### Tier 1: Nuclear translocation of the transcription factor Nrf2 in endothelial cells

Recent studies with endothelial cells directly exposed to DEPM demonstrated that these particles induce oxidative stress (*tier 1*) and an inflammatory response (*tier 2*) [36, 37]. To evaluate the potential of DEPM to induce the nuclear translocation of the Nrf2, tetracultures were exposed to 240 ng/cm^2^ of DEPM via an aerosol exposure system (Vitrocell™) and evaluated at 3, 4 and 5 h after exposure using confocal laser scanning microscopy. As control, tetracultures that were kept in the aerosol chamber for the same exposure time but with the particle generator in stand-by mode (constant air flow but no particles) served as control.

**Fig. 6:**
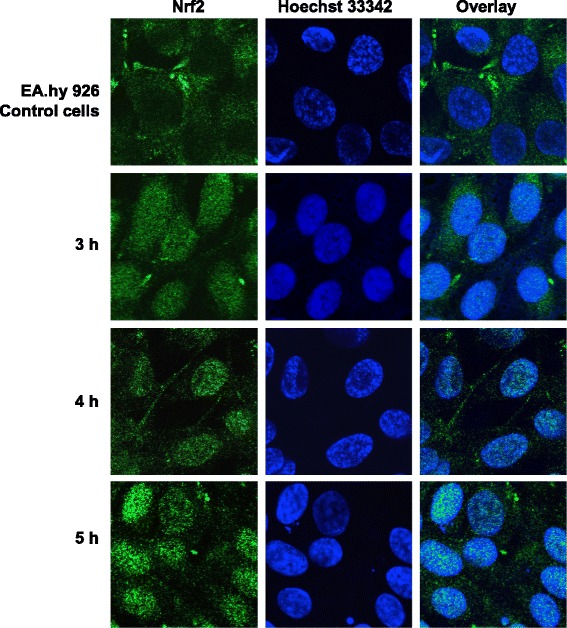
Potential of DEPM to induce nuclear translocation of Nrf2 in endothelial cells after different incubation times. Tetracultures were exposed to 240 ng/cm^2^ of DEPM. Nrf2 translocation was evaluated for cells indirectly exposed to DEPM at 3, 4 and 5 h after exposure. Cells exposed for the same time but without particles in the air stream were used as controls for the fluorescence signals. Cells were fixed and stained for nucleus (Hoechst 33342) and anti-Nrf2 (green). Images were analyzed using a Zeiss LSM 510 META. At 3 h after exposure, first cells showed nuclear translocation. At 4 h after exposure, nuclear translocation was quite obvious in the majority of the cell population. At 5 h after exposure almost every endothelial cells showed strong colocalization of Nrf2 with the nucleus (counterstained in blue). In control EA.hy 926 cells, no nuclear translocation was observed at any time-point (compare with the representative control image)

Nrf2 translocation could already be detected in some cells 3 h after exposure (Fig. 6). At 4 h after exposure, the nuclear translocation of Nrf2 was detected in the majority of the cells. Finally, at 5 h after the exposure almost all endothelial cells showed strong co-localization of Nrf2 with the nucleus. In control EA.hy 926 cells without particle exposure, no nuclear translocation was observed at any time-point (Fig. 6).

#### Tier 2: Oxidative stress in endothelial cells

Recent studies with endothelial cells directly exposed to DEPM demonstrated that these particles induce an inflammatory response [36, 37]. Nrf2 regulates the expression of key genes critical for cellular defense against oxidative stress, such as *NQO1*, *SOD1, GST1* and *HMOX1*. The potential upregulation of these genes as well as the upregulation of the gene (*NEF2*) coding for Nrf2 was evaluated by qRT-PCR. After exposure of tetracultures to realistic doses of DEPM, mRNA levels of markers representative for the 3 tiers of the hierarchical oxidative stress response were quantified in endothelial cells (Table 1).

Regarding the mRNA levels of the *tier 1* indicators *NQO1*, *SOD1, GST1* and *HMOX1* and the tier 2 indicators *ICAM-1* and *IL-6,* no difference in mRNA levels compared to the control cells without particles could be seen at all time-points. *VCAM1* and *E-Selectin* were below the limit of detection in exposed cultures and controls (Table 1). For the transcription factor NF_k_B, which is involved in the modulation of the pathways in *tier 2*, no increase in mRNA level could be detected for the cells exposed to 80 ng/cm^2^ of DEPM. 48 h after the exposure to 240 ng/cm^2^, the expression of *NF*_*k*_ *B* was slightly (1.2 ± 0.07) but significantly increased compared to untreated controls (*P* < 0.05) (Table 1).

Similarly to the results of the evaluated marker genes for endothelial inflammation, none of the evaluated second messengers, such as IL-6, IL-8, TNF-a, GM-CSF or MCP-1, analyzed in the tetraculture undernatant as indicators for the onset of inflammation, was significantly increased after DEPM exposure compared to untreated cultures (Table 2).

**Table 2:** Second messengers in cell culture undernatants of the tetraculture after DEPM exposure for 6, 24 and 48 h as markers for *tier 2*

			80 ng/cm^2^			240 ng/cm^2^		
Cytokine	Function		6 h	24 h	48 h	6 h	24 h	48 h
			pg/mL	pg/mL	pg/mL	pg/mL	pg/mL	pg/mL
L-6	Hematopoiesis, thrombopoiesis; induces T cell proliferation and differentiation; Variable effects on cytokine release by monocytes	control	19.5 ± 5.7	34.3 ± 6.3	29.8 ± 7.5	19.4 ± 3.7	30.9 ± 10.1	22.7 ± 5.7
		exposed	27.9 ± 5.2	30.3 ± 4.5	39.6 ± 14.3	19.2 ± 6.0	25.5 ± 3.4	29.9 ± 6.7
IL-8	Chemotactic and inflammatory cytokine; activates neutrophils inducing chemotaxis, exocytosis and the respiratory burst; elicits neutrophil accumulation at the site of inflammation	control	5815 ± 610	6183 ± 530	6236 ± 665	4719 ± 484	6163 ± 540	5531 ± 693
		exposed	4905 ± 754	5218 ± 681	4908 ± 673	4866 ± 807	5092 ± 596	4905 ± 785
TNF-α	Monocyte activation, cytokine and prostaglandin upregulation; Endothelial cell adhesion molecule and cytokine release upregulation	control	4.0 ± 1.7	3.9 ± 0.4	10.3 ± 2.9	2.9 ± 0.7	4.6 ± 0.8	11.7 ± 2.8
		exposed	4.0 ± 1.4	6.2 ± 1.1	6.1 ± 0.9	3.1 ± 1.3	6.4 ± 1.6	8.0 ± 1.7
GM-CSF	Granulocyte and monocyte maturation; leukocyte prostaglandin release; DC maturation; pulmonary surfactant turnover	control	10.9 ± 2.7	23.7 ± 1.4	81.0 ± 12.8	6.8 ± 1.5	23.9 ± 2.5	82.4 ± 5.2
		exposed	6.3 ± 1.5	24.6 ± 4.0	78.2 ± 12.6	23.0 ± 16.6	23.4 ± 2.5	83.7 ± 10.4
MCP-1	Plays a crucial role in initiating atherosclerosis by recruiting macrophages and monocytes to the vessel wall	control	4832 ± 1181	6000 ± 1189	10545 ± 1308	4832 ± 1181	7428 ± 1087	8985 ± 942
		exposed	4697 ± 1187	7814 ± 493	8708 ± 1187	4315 ± 621	6974 ± 473	8392 ± 1129

Tetracultures were exposed to 80 ng/cm^2^ and 240 ng/cm^2^ DEPM. After exposure, undernatants were collected and analyzed for the 30 different second messengers using a MESO QuickPlex SQ120. In table 2 are only reported those that were of relevance for early endothelial activation. Data represents the mean of at least four independent experiments ± SEM

#### Tier 3 related effects in endothelial cells

Regarding the concept of the hierarchical oxidative stress response, in *tier 3* the oxidative stress overwhelms cellular defense capacities, resulting in cellular death [20].

Environmental particles as well as some chemicals were shown to interfere with programmed cell death, which may result in an unintended apoptotic activity of otherwise healthy cells and tissue. Therefore, expression of the pro-apoptotic key genes *FAS* and *CASP7* was also evaluated.

The endothelial cells of the tetraculture showed no increase in the expression of *FAS* mRNA after the indirect exposure to 80 and 240 ng/cm^2^ at any of the evaluated time-points. *CASP7* mRNA was significantly upregulated in endothelial cells that were indirectly exposed to 240 ng/cm^2^ at 48 h after the exposure (1.2 ± 0.04; *P* < 0.05 compared to untreated controls). The other samples were at the same level as the control cells (6 h: 0.96 ± 0.05; 24 h: 1.00 ± 0.05) (Table 1).

### DEPM exposure as potential inducer of *CYP1A1* expression in the endothelial cells

**Fig. 7:**
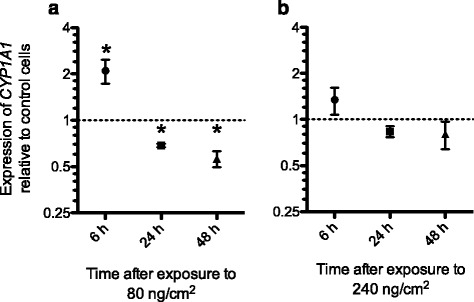
Differential gene expression profile of *CYP1A1* in endothelial cells at different incubation time after DEPM exposure of the tetraculture system. The endothelial cells of the tetraculture showed a significant increase for *CYP1A1* mRNA 6 h after the indirect exposure to 80 ng/cm^2^ of DEPM (**a**). No significant change was observed for cells exposed to 240 ng/cm^2^ of DEPM (**b**). Results were normalized to untreated control cells (dotted line). Data represents the mean of four independent experiments ± SEM. Asterisks indicate significant differences compared to untreated control cells (*P* < 0.05)

Organic chemicals such as B[a]P can adsorb to the outer shell of DEPM [21]. In analogy to the experiments described above, the potential of DEPM to induce mRNA expression of *CYP1A1* was studied. We found a significant increase (1.85 ± 0.37 fold) of *CYP1A1* expression compared to unexposed cells after 6 h exposure to 80 ng/cm^2^ of DEPM (*P* < 0.05), while levels were significantly reduced at later time points (24 h: 0.65 ± 0.03; 48 h: 0.56 ± 0.06) (*P* < 0.05) (Fig. 7). In contrast to the results obtained with 80 ng/cm^2^, 240 ng/cm^2^ did not lead to significantly enhanced *CYP1A1* mRNA levels.

Despite the significantly altered expression of *CYP1A1* mRNA a clear change in the subcellular distribution of the transcription factor AhR could not be observed (data not shown).

## Discussion

During recent years, the alveolar endothelium gained increased attention as secondary target structure for the effects of DEPM [38]. Impaired endothelial cell function is characterized by reduced dilatory properties and an increased expression of surface adhesion molecules, such as VCAM1 or E-Selectin, which are critical for adhesion of inflammatory cells and induction of an inflammation [39, 40]. Such endothelial inflammation may later on lead to severe diseases, like atherosclerosis and myocardial infarction [24]. In order to explain the adverse effects of particulate air pollution on human health, the concept of the hierarchical oxidative stress response was developed [23] (Fig. 1) and it was proposed to be applicable to the endothelium, too [41]. In the present study, the main focus was to study the effects in endothelial cells of a novel alveolar tetraculture model after exposure to in vivo relevant DEPM doses.

In this study tetracultures of alveolar epithelial cells (A549), which are able to produce and secrete surfactant, mast cells, that are also present on the alveolar surface [42], differentiated THP-1 cells and EA.hy 926 cells [30] were exposed to different doses of DEPM (SRM2975) at the air-liquid-interface. Compared to many in vitro studies with DEPM and monocultures of endothelial cells that were conducted in the past [43], the present study is the first approach to combine a complex 3D tetraculture in vitro system with native aerosol exposure equipment to mimic the exposure situation in vivo as closely as possible and to study the indirect effects of exhaust particles on the endothelial cells of the alveolar barrier. DEPM exposure did not lead to the mRNA expression of relevant markers for endothelial inflammation such as *ICAM-1* or *E-selectin*. In addition, we observed neither a significant change in the expression levels of the genes relevant for antioxidant defense, such as *HMOX1* or *SOD1*, nor the release of pro-inflammatory second messengers, such as IL-6, IL-8, TNF-α, GM-CSF and MCP-1*.* However, DEPM exposure led to strong nuclear translocation of the transcription factor Nrf2, comparable to endothelial cells treated with an oxidative stress inducer in monoculture (Additional file 1: Figure S1), and expression of *CYP1A1* mRNA in the endothelial cells of the tetraculture. Overall, the applied doses of DEPM did not significantly affect cellular viability or inflammatory status at any of the monitored time-points under assay conditions (see also Additional file 2: Table S2).

The results for cellular viability are in line with those of recently published studies where the effects of catalyzed and non-catalyzed diesel on lung epithelial coculture models exposed at the ALI were evaluated using comparable doses [44].

It is however important to emphasize that cellular viability and mediators can give only an overview on the status of the whole tetraculture system including all cell lines. Since our focus was specifically laid to the processes and changes in the endothelial cells of the tetraculture as indirect effect of DEPM exposure, important marker genes for anti-oxidant defense and endothelial inflammation were analyzed in these cells only. However, no significant changes for these markers towards inflammation could be detected at any time-point.

For our study, the DEPM particles from NIST were used, since they are well characterized for their chemical composition and represent a sort of particles that has been widely used in the past as model particles to evaluate the effects of combustion-derived PM on human health. However, these particles were collected from a heavy-duty diesel forklift without any type of particle filtering system, so that the obtained results are not fully transferable to nowadays traffic conditions, where modern exhaust filter systems are mandatory for new diesel powered cars in Europe. It is interesting to notice that the expected peak around 100 nm, which is typical of the exhaust of diesel engine, is not present, but the most representative size is around 450-500 nm [45]. We can only hypothesize this could be due to the collection or preparation processes that could have favored the aggregation of smaller particles into larger aggregates. Regarding the observed effects, it is also important to emphasize that the effects observed here are caused only by the “solid phase” of the exhaust since the gaseous phase was not present. However, in respect to the largely growing body of evidence, the solid phase of diesel exhaust represented by DEPM, is likely to play the major role for the observed cardiovascular effects (reviewed in [43]).

Nevertheless, the SRM2975 is still a good model regarding the missing implementation of strict exhaust filter systems for current diesel powered ships [46], which are also considered to be responsible for significant health effects on the human cardiovascular system (reviewed in [47]).

The presence of metals was proposed to be an important factor for the potential of ultrafine particles to induce oxidative stress in cellular systems [20]. The presented results for the low potential of SRM2975 to induce endothelial activation are in line with those of other investigators from in vivo and in vitro experiments evaluating the acute effects after exposure to DEPM [48–50].

Even though the amount of metals that is present in DEPM from NIST may be too low to exhibit a significant anti-oxidant defense that follows the model of the hierarchical oxidative stress response, we observed the activation and translocation of the transcription factor Nrf2 clearly involved in antioxidant defense. In line, DEPM extracts are known to activate the AhR and induce intracellular ROS generation, e.g. via CYP enzymes. Interestingly, there is growing evidence on molecular interaction between AhR and Nrf2 [51]. Investigators assume that the AhR, once being activated by a PAH ligand, may directly interact with the Nrf2 inhibitor Keap1, leading to nuclear accumulation and potential expression of target genes [51]. However, others speculated that metabolites of PAHs may contribute to the activation of Nrf2 [52]. The increased expression of *CYP1A1* early after exposure may have led to enhanced clearance of potential Nrf2 ligands, avoiding significant elevation of Nrf2 target gene levels.

Our results show that the exposure to low amounts of DEPM leads to significant upregulation of *CYP1A1* mRNA early after exposure followed by a significant downregulation at later time-points. In addition also other investigators reported about the potential of DEPM to interfere with the expression level of *Cyp1a1* in rat alveolar cells [53]. Others speculated that such a downregulation after exposure to exhaust components could represent a cytoprotective response [54], preventing the cells from toxic metabolites. It seems reasonable to assume that this effect is stronger for the higher dose of DEPM, preventing a significant change of *CYP1A1* mRNA for the evaluated time-points.

The significant changes at 48 h after exposure in the expression levels of the transcription factor *NF*_*k*_ *B* and the chaperon *HSP70* point towards a delayed response after the acute exposure to DEPM. NF_k_B is known to be involved in the upregulation of cytokines and the transcription of cellular adhesion molecules, such as ICAM-1 and VCAM1 at the beginning of an inflammation [55]. Data from in vivo experiments with rodents suggest that upregulation of *NF*_*k*_ *B* expression in the endothelium play a central role in inflammatory vascular diseases, such as atherosclerosis, after long-term exposure [56, 57]. HSP70 is used as general marker for cellular stress [58], and it was reported to contribute to endothelial dysfunction and cardiovascular disease [59]. In addition, it was shown that expression of *HSP70* can be upregulated in response to PAHs, such as B[a]P, that can also be found in DEPM [60]. Also the upregulation of *CASP7* mRNA 48 h after the exposure is supporting the hypothesis of a delayed response in the endothelium following indirect exposure. The upregulation of the evaluated marker genes in the endothelial cells 48 h after the exposure of tetracultures therefore is likely a sign of a delayed stress response in the endothelial cells.

The doses of DEPM that were used in the present study can be considered to represent situations of acute smog episodes, such as recently reported for Bejing, China, where levels for PM_2.5_ of 800 μg/m^3^ air were recorded [61]. Even though this represents an extreme scenario resulting in a possible particle overload of the alveolar epithelium [62], the applied doses used in our experimental setup could be inhaled by individuals within a single day, in contrast to other in vitro studies using DEPM where the applied doses exceed even the maximum of what humans could acquire during their lifetimes. These high particle loads are several orders of magnitude lower compared to what was used for studies with DEPM in submerged exposure (reviewed in [25]). The presented 3D model system could serve to further investigate the effects of particulate air pollution on the endothelium and may help to understand involved cellular mechanisms that finally lead to inflammation and contribute to cardiovascular disease in more detail.

## Conclusion

In the present study, we demonstrated the use of a complex 3D tetraculture system together with a state-of-the-art aerosol exposure equipment to study the effects of in vivo relevant doses of DEPM on endothelial cells in vitro. To the best of our knowledge, this study is the first that focuses on indirect effects of DEPM on endothelial cells of the alveolar barrier in vitro. Exposure to DEPM led to significant activation and nuclear translocation of the transcription factor Nrf2 in endothelial cells. The considerably low doses of DEPM had a low but measurable effect, which is in line with recent data from in vivo studies.

## Methods

### Reagents

All reagents, unless otherwise specified, were purchased from Sigma-Aldrich (Deisenhofen, Germany). Cell culture media were purchased from Invitrogen (the Netherlands), fetal bovine serum (FCS) was obtained from Biochrom (Berlin, Germany).

### Cultivation of cells and preparation of tetracultures

Cells were grown in T75 flasks and trypsinized twice a week. Medium (inserts and cell culture flasks) was changed every other day. Cells were maintained in a humidified atmosphere with 5% CO_2_ at 37 °C and tested regularly for contamination by mycoplasma. For experiments cells were seeded at specified densities: A549 1.2 x 10^5^ cells/cm^2^; EA.hy 926 2.4 x 10^5^ cells/cm^2^; THP-1 2.4 x 10^5^ cells/cm^2^; HMC1 1.2 x 10^5^ cells/cm^2^ on BD Falcon cell culture inserts (surface area of 4.2 cm^2^; 1 μm pore size; high pore density PET membranes for 6-well plates; BD Biosciences, Basel, Switzerland) and grown until confluency as described in the following:

EA.hy 926 endothelial cells were seeded on inverted transwell inserts (2.4 × 10^5^ cells/cm^2^). Upon attachment on the basolateral side of the transwell insert the plate with the transwell inserts was turned back to its original orientation before the A549 cells were seeded inside the transwell. Epithelial and endothelial cells were grown for four days at 37 °C and 5% in a humidified incubator. On day 3 THP-1 cells were stimulated to differentiate into macrophage-like cells by addition of phorbol-12-myristat-13-acetat (as previously described in [30]). On day 4, THP-1 cells and HMC-1 cells were added into the inserts with A549 and EA.hy 926 cells to complete the tetraculture system. Inserts were placed in BD Falcon tissue culture plates (6-well plates; BD Biosciences) with 2 mL medium in the upper and 2 mL in the lower compartment. The medium for the exposure of the tetraculture contained only 1% FBS to avoid extensive proliferation of HMC-1 cells (Table 3). Upon attachment of THP-1 and HMC-1 cells (generally after 4 h) the medium was removed from the upper compartment and the tetraculture was cultivated at the ALI for 24 h prior to exposure.

**Table 3:** Medium conditions for mono- and cocultures. Unless further mentioned, mono- and cocultures were handled in the absence of antibiotic and antimycotic agents. All media contained Glutamax instead of L-glutamine

Monocultures			
Cell Line	Medium	Serum	Supplements
A549	Dulbecco’s Modified Eagle’s Medium (DMEM)	10% (v/v) Fetal Bovine Serum Gold	
THP-1	Roswell Park Memorial Institute (RPMI) 1640		25 mM HEPES; 50 μM β-mercaptoethanol
HMC-1	Iscove’s Modified Dulbecco’s Medium (IMDM)		25 mM HEPES; 1,2 mM α-thioglycerol
EA.hy 926	Dulbecco’s Modified Eagle’s Medium (DMEM)		25 mM HEPES
Tetraculture			
75% HEPES-buffered DMEM; 15% RPMI; 10% IMDM		1% (v/v) Fetal Bovine Serum Gold	25 mM HEPES

### Aerosol exposure

The Vitrocell™ aerosol exposure device (Vitrocell™ 6/3 CF stainless, Vitrocell™ Systems, Germany) (Vitrocell™, Taufkirchen, Germany) was used for dynamic delivery and exposure of cells to aerosolized DEPM. The uniformity of the particle population was confirmed by scanning electron microscopy (SEM).

Briefly, the Vitrocell™ aerosol exposure device contains three exposure chambers, which holds one separate insert each, allowing simultaneous exposures of 3 Transwell™ inserts. In order to keep the cells viable, the module is equipped with a heated water jacket at a steady temperature of 37 °C. For the exposure, cell culture inserts are placed into the exposure chambers containing the culture medium.

The aerosol is generated from powder by a dry-aerosol generator with a rotating steel brush (RBG 1000 PALAS, Karlsruhe, Germany) and was delivered through a trumpet device at a low flow rate (5 ± 0.1 mL/min/module) for a defined time of exposure to the modules. For exposure, the rotating steel brush was operated at 1200 rpm and the speed of the piston, which forwarded the DEPM towards the brush, was set to 30 mm/h. Cells were exposed for 1 min 8 s for 40 ng/cm^2^, 2 min 17 s for 80 ng/cm^2^, or 6 min 52 s for 240 ng/cm^2^. After exposure, transwells with tetracultures were placed in fresh medium (as described recently [30]) and further incubated for another 6, 24 or 48 h.

Before harvesting of endothelial cells or sampling of undernatants, all Transwell™ inserts were routinely checked by light microscopy to verify the integrity of the cell layer. All inserts showed no abnormalities of the cellular layers (data not shown).

### Aerosol particle counting

For the aerosol particle count two different systems were used.

In order to measure the range from 250 nm to 2 mm, we used an optical particle counter (GRIMM Aerosol EDM 164 portable fine dust aerosol spectrometer). The instrument is built according to measuring standards EN12341, EN14907, US-EPA and GOST-R. A continuous flow of sample air (1.2 L/min ± 5% continuously regulated) is led into the measuring cell where the dust particles are measured by the physical principle of orthogonal light scattering. Every single particle is detected and classified into defined particle sizes based on the intensity of the scattering light signal. The laser spectrometer detects all airborne particles in a size range from 0.25 μm up to 32 μm in real-time, with a particle size distribution in 31 channels.

In order to measure the nano range from 5 nm to 300 nm, which is not visible with standard optical counter, we used a Sequential Mobility Particle Sizer and Counter (SMPS + C) by GRIMM Aerosol, which combines a Condensation Particle Counter (CPC) and a Differential Mobility Analyser (DMA). Briefly, the DMA allows the selection of different size classes which are sequentially introduced in the CPC, in which the particles are grown by condensation of butanol vapours becoming suitable for optical detection.

### Scanning electron microscopy (SEM)

Empty Transwell™ inserts were exposed for 30 min as described for Transwell™ inserts containing the tetraculture system. Afterwards samples were metallized with a 20 nm gold film under vacuum. Scanning electron microscopy was done with a FIB-SEM (FEI, Eindhoven, The Netherlands) at 25 kV and 25 mA.

### NanoSIMS50 analysis

Samples were analyzed with a NanoSIMS50 (Cameca, Courbevoie, France) using a Cs^+^ primary source (8 keV), rasterizing the surface of the sample (-8 keV) with a raster between 40 x 40 μm and 20 x 20 μm to generate secondary negative ions. The energy of the impact of the primary beam was 16 keV with an intensity range of 1.0-0.8 pA. In these conditions the probe-working diameter was in the range of 80–100 nm. The masses studied simultaneously in multicollection mode were: ^31^P^-^ (m = 30.97376), ^54^Cr (m = 53.9388804), ^58^Ni (m = 57.9353429), ^67^Cu (m = 66.9277303) and ^64^Zi (m = 63.9291422). Images were recorded in a pixel format of 256 x 256 image points with a counting time of 20 ms per pixel.

### Dosimetry approach for DEPM

Since the crystal surface of the quartz microbalances provided by Vitrocell was found to be not compatible with the lightweight and electrostatically charged DEPM, we established an alternative method based on the approach published by Rudd and Strom [63] to evaluate the deposition of DEPM per cm^2^. The method uses the absorption of DEPM at 750 nm. To evaluate the amount of particles per cm^2^, Transwell™ inserts were pre-wetted with 1 mL of 0.1 N NaOH for 1 min in order to allow the membrane to adsorb some liquid to simulate a slightly wet cellular surface. Subsequently, these inserts were exposed for different times and subsequently washed with 200 μL of 0.1 N NaOH to collect all particles from the membrane. The absorption of these particle suspensions was then measured with a spectrophotometer and compared to a DEPM standard curve to calculate the mass of deposited particles. Particle deposition for DEPM was found to be uniform for up to 20 min of exposure, resulting in a deposition maximum of 600 ng/cm^2^. The results were used to extrapolate the exposure times needed to expose tetracultures to 40, 80 and 240 ng/cm^2^ of DEPM.

### Cell labeling and fixation for confocal microcopy

The cells were washed twice in PBS and subsequently fixed for 10 min at -20 °C in 100% ice-cold methanol. After fixation, cells were washed again and the fixed cells were incubated for 30 min with 10% FBS in PBS (w/v) to block unspecific bindings. After blocking, cells were incubated with primary and secondary antibodies for 60 min each at room temperature in the dark.

Antibodies (Table 4) were diluted in PBS containing 1% FBS. Nuclei were counterstained with Hoechst 33342.

**Table 4:** List of antibodies used for immunohistochemistry labeling

Target	Species	Dilution	Reference
Primary antibodies			
Nrf2	Rabbit	1:100	ab31163; Abcam, UK
AhR	Rabbit	1:200	sc-5579; H-211; Santa Cruz, Heidelberg, Germany
Secondary antibodies			
Anti-rabbit	Goat	1:2000	AS09 633; Agrisera, Vännas, Sweden

### Confocal Microscopy and Image Restoration

A Zeiss LSM 510 Meta with an inverted Zeiss microscope (Axiovert 200 M, Lasers: HeNe 633 nm, HeNe 543 nm, Ar 488 nm and Diode 405 nm; Zeiss, Jena, Germany) was used. Image processing and visualization was done using the Zeiss Software ZEN 2011 and Image*J* (http://rsbweb.nih.gov/ij/).

### qRT-PCR with endothelial cells from tetracultures

In order to separate the endothelial cells from the transwell membrane, the lower compartment of the transwells was washed twice with 1 mL of PBS. Afterwards, EA.hy 926 cells were detached by using 800 μL of trypsin/EDTA for 5 min. The success of the procedure was verified using light microscopy (not shown). Subsequently after detaching, the endothelial cells were washed twice with PBS and centrifuged for 2 min at 800 g and supernatants were discarded. Total RNA was isolated from cell pellets with RNeasy Mini Kit (Qiagen, Leusden, Netherlands), following the manufacturer’s instructions. RNA integrity control was assessed with the RNA Nano 6000 assay (Agilent Technologies, Diegem, Belgium) using a 2100 Bioanalyzer (Agilent Technologies, Diegem, Belgium). All RNA samples displayed RINs (RNA integrity number) above 8. Concentration and purity determination of the RNA were performed by using a Nanodrop ND1000 spectrophotometer (Thermo Scientific, Villebon-sur-Yvette, France). Reverse transcription was performed with the following reagents: M-MuLV Reverse Transcriptase (RNase H), murine RNase inhibitor (both obtained from New England Biolabs, Ipswich, MA, USA) and random primers (Invitrogen, Carlsbad, NM, USA) following the manufacturer instructions. 20 μL final volume containing 5 μg of total RNA were used to perform the reverse transcription. Four reference genes *B2M*, *HPRT*, *SDHA*, *YWHAZ* (Table 5) [64] primers pairs were ordered from Eurogentec (Liege, Belgium). The reference genes were validated as stable candidates between the conditions using GeNorm in the Biogazelle qBase PLUS software. Gene expression was evaluated using a real-time PCR system (Life technologies) together with mesa green low rox real-time PCR kits (Eurogentec, Liège, Belgium). PCR were performed in 25 μL with final concentrations as follows: 1X MasterMix, 100 nM for each primers except for *CYP1B1* where 300 nM forward and 100 nM reverse primers were used, 0.4 ng/μL cDNA. Each sample was performed in triplicate. Template control and RT- control samples were added to each plate. Thermal cycling conditions were: initial 5 min denaturation at 95 °C, followed by 45 cycles of 15 s at 95 °C and 1 min at 60 °C, and a final dissociation step (melting curve) was used to determine the primer specificity by revealing the presence of a single peak. PCR efficiency was performed using decreasing five-fold dilution from cDNA pool (from 25 ng to 0.04 ng and no template control). Contamination by genomic DNA was verified by using total RNA as sample for PCR. The relative gene expression was calculated taking into account gene-specific PCR efficiency [65, 66] using the Biogazelle qbase PLUS software 2.5 software with the ^ΔΔ^CT method. Table 5 summarizes the primers that were used in this study.

**Table 5:** Summary of the primer sequences that were used for qRT-PCR experiments with the endothelial cells of the tetraculture

Gene	Primer F	Primer R	Reference
*HMOX1*	TTCTCCGATGGGTCCTTACACT	GGCATAAAGCCCTACAGCAACT	[44]
*CASP7*	CGGTCCTCGTTTGTACCGTC	GGTGGTCTTGATGGATCGCA	[44]
*SOD1*	GTGCAGGTCCTCACTTTAAT	CTTTGTCAGCAGTCACATTG	[44]
*FAS*	AGCTTGGTCTAGAGTGAAAA	GAGGCAGAATCATGAGATAT	[44]
*NF*_*k*_ *B*	GCTTGTAGGAAAGGACTGCC	GTTGTTGTTGGTCTGGATGC	[71]
*CYP1A1*	GGAGGCCTTCATCCTGGAGA	CCTCCCAGCGGGCAACGGTC	[72]
*IL-6*	GGAGACTTGCCTGGTGAAAA	GTTGGGTCAGGGGTGGTTAT	
*NEF2*	ACATTGAGCAAGTTTGGGAG	TGTGGACTACAGTTACCTAC	
*NQO1*	GGAGAGTTTGCTTACACTTACGC	TTCTCCAGGCGTTTCTTCCA	
*ICAM-1*	GCAAGGTGACCGTGAATGT	GCATAAAGCCCTACAGCAAC	
*GST1*	ACAGTTGTACAAGTTGCAGGATG	TGCCAAAGAGATTGTGCTTG	
*HSP70*	CCTACTCCGACAACCAACCC	GGTGATCTTGTTGGCCTTGC	
*HMOX2*	GGGAAAGGAGACATGCGTAA	CAAGAGTCCAGCAGCTAGGG	
*E-Selectin*	ACCTCCACGGAAGCTATGACT	CAGACCCACACATTGTTGACTT	
*VCAM1*	CTTAAAATGCCTGGGAAGATGGT	GTCAATGAGACGGAGTCACCAAT	
*B2M*	TGCTGTCTCCATGTTTGATGTATCT	TCTCTGCTCCCCACCTCTAAGT	[64]
*HPRT1*	TGACACTGGCAAAACAATGCA	GGTCCTTTTCACCAGCAAGCT	[64]
*YWHAZ*	ACTTTTGGTACATTGTGGCTTCAA	CCGCCAGGACAAACCAGTAT	[64]
*SDHA*	TGGGAACAAGAGGGCATCTG	CCACCACTGCATCAAATTCATG	[64]

Unless further mentioned, qRT-PCR primer were designed for the experiments of this study

### Evaluation of the potential of EA.hy 926 to react to oxidative stress

For the evaluation of the ability of EA.hy 926 cells to show nuclear translocation of Nrf2 and corresponding target gene expression they were exposed to inducers of oxidative stress in complete cell culture medium. To visualize the Nrf2 translocation, EA.hy 926 cells were exposed to 20 mM 2,2’-azobis-2-methylpropanimidamide-dihydrochloride (AAPH) for 2 h (Additional file 1: Figure S1). The expression of the Nrf2 target genes was investigated on EA.hy 926 cells exposed to three concentrations of AAPH (4, 20, 100 mM) and *tert*-butylhydroquinone (tBHQ) (10, 40, 80 μM) for 6 and 24 h (Additional file 2: Table S1). As negative control, cells were kept in complete cell culture medium without AAPH or tBHQ.

The methods used in these experiments with EA.hy 926 cells (cell labeling and fixation for confocal microscopy, qRT-PCR) are the same as the ones described for the tetraculture system.

### Cytokine measurements

Second messengers in cell culture undernatants of the tetraculture were analyzed by using the “V-PLEX Human Cytokine 30-Plex Kit“ from Mesoscale Diagnostics, Rockville, USA for the evaluation of levels of Eotaxin, Eotaxin-3, GM-CSF, IFN-γ, IL-10, IL-12/IL-23p40, IL-12p70, IL-13, IL-15, IL-16, IL-17A, IL-1α, IL-1β, IL-2, IL-4, IL-5, IL-6, IL-7, IL-8, IL-8 (HA), IP-10, MCP-1, MCP-4, MDC, MIP-1α, MIP-1β, TARC, TNF-α, TNF-β and VEGF-A. In Table 2 the secondary messengers that were above the limit of detection in the medium of treated tetracultures are reported (see also Additional file 3 Table S2). Sample analysis was performed using MESO QuickPlex SQ120 (Mesoscale Diagnostics) according to the manual provided by the manufacturer.

### Resazurin metabolism assay for the 3D tetraculture

Transwell™ inserts containing the 3D tetracultures were washed twice with PBS. Afterwards, 2 mL of tetraculture medium containing 400 μM resazurin were added to the upper and the lower compartment. Cells were incubated for one hour at 37 °C and 5% CO_2_. After the incubation, aliquots of 500 μL were taken from the upper and the lower compartment and transferred into a 12 well plate to measure separately the fluorescence for both compartments (inside and outside of the Transwell™ insert). Fluorescence reading was done with a multi-mode microplate reader (ex: 530 nm, em: 590 nm; Biotek, Germany).

### Statistics

Data represents the mean of at least three, respectively four independent experiments ± standard error of mean. Statistical analysis was done using SPSS, version 19 (IBM, Mainz, Germany). Statistical comparison of the means was performed by ANOVA, followed by the Tukey posthoc test. In figures, significant differences are indicated by asterisks (*) (*P* < 0.05). In tables, significant differences are indicated by bold letters (*P* < 0.05).

## Additional files

Additional file 1: Figure S1. Nuclear translocation of Nrf2 in endothelial EA.hy 926 cells in monoculture after treatment with 20 mM AAPH for 2 h. EA.hy 926 cells were exposed to 20 mM AAPH for 2 h. Cells kept in complete medium without AAPH served as solvent control. Nrf2 translocation was evaluated for 2 h after treatment. Cells were stained with anti-Nrf2 antibody (*green*), nuclei are counterstained with Hoechst 33342 (*blue*). Images were analyzed using a Zeiss LSM 510 META. 2 h after exposure almost every endothelial cells shows strong colocalization of Nrf2 with the nucleus (*red arrows*). In control EA.hy 926 cells, no nuclear translocation was observed (compare *white arrows*). (PDF 13756 kb)
Additional file 2: Table S1. Differential gene expression observed in EA.hy 926 cells exposed for 6 and 24 h to AAPH and tBHQ at different concentrations. The response factors are ratios between the gene expression levels (calculated relatively to the housekeeping genes) after exposure related to their respective solvent control (mean ± SEM, n = 3). Bold letters indicate significant differences between the evaluated time-points (*P* < 0.05 with a fold change greater than 2). (PDF 15 kb)
Additional file 3: Table S2. Secretion of second messengers after exposure of the tetraculture to different doses of DEPM at different time-points. Tetracultures were exposed to 80 ng/cm^2^ or 240 ng/cm^2^ of diesel exhaust particles. Aliquots of the undernatant were taken at 6, 24 and 48 h after exposure and stored upon analysis at -80 °C. The level of secreted second messengers was evaluated using a multiplexed assay from Mesoscale Diagnostics for pro-inflammatory cytokines (A), cytokines (B) and chemokines (C). Tetracultures that were kept in the aerosol chamber for the same exposure time but with the particle generator in stand-by mode served as control. Data represents the mean of at least four Transwell™ inserts ± SEM. (PDF 125 kb)

## Abbreviations

B[a]P: Benzo[a]pyrene
Cr: Chromium
Cu: Copper
DEPM: Diesel exhaust particulate matter
NanoSIMS50: Secondary ion mass spectrometry
Ni: Nickel
PAHs: Polycyclic aromatic hydrocarbons
PM: Particulate matter
ROS: Reactive oxygen species
SEM: Scanning electron microscopy
Zn: Zinc
