# Supplementary figures and images for: Endothelial responses of the alveolar barrier in vitro in a dose-controlled exposure to diesel exhaust particulate matter
Source: Part Fibre Toxicol. 2017 Mar 6;14:7. doi: 10.1186/s12989-017-0186-4 (PMC5339948; doi:10.1186/s12989-017-0186-4)

**Nrf2**

**Hoechst 33342**

**Overlay**

**EA.hy 926  
control  
cells**

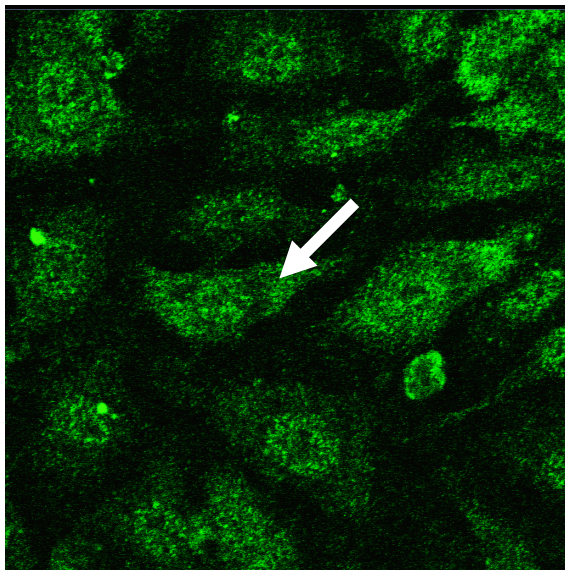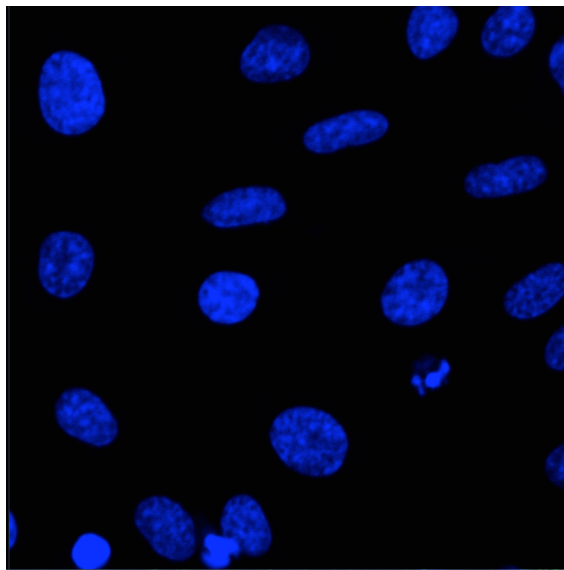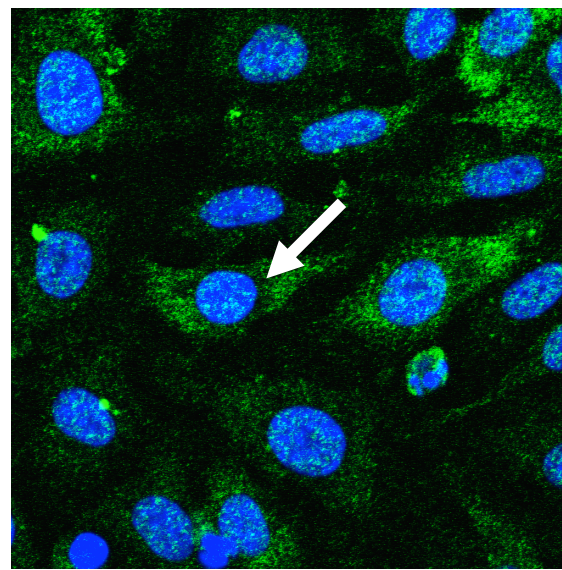

**2 h  
20 mM  
AAPH**

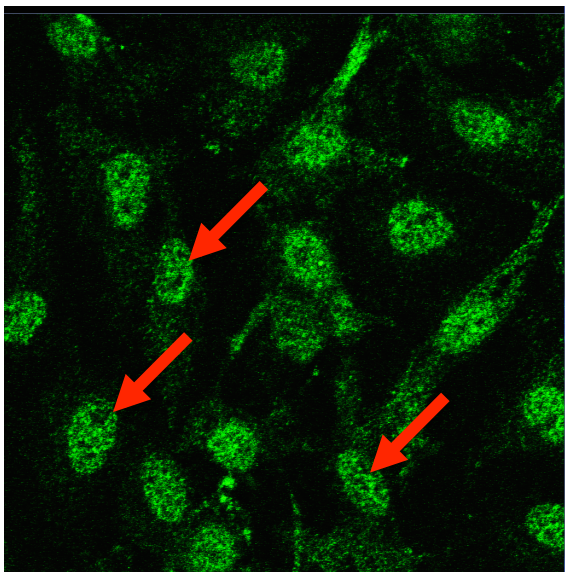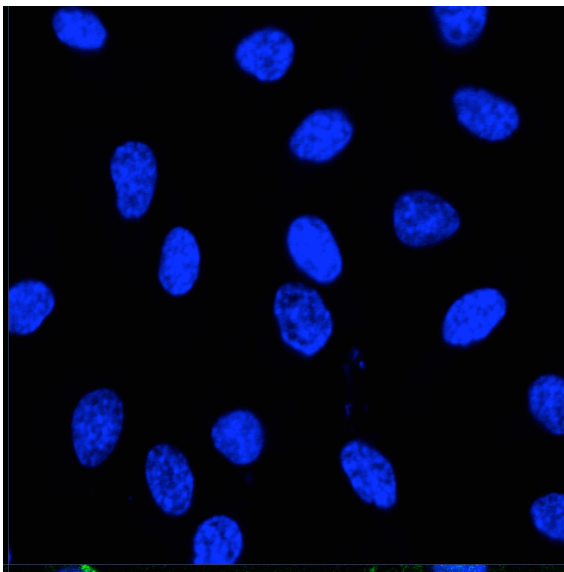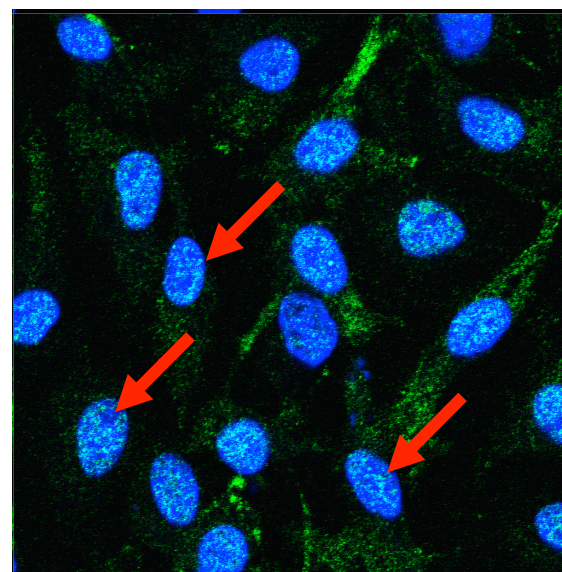

Supplement: Additional file 1: Figure S1. — Nuclear translocation of Nrf2 in endothelial EA.hy 926 cells in monoculture after treatment with 20 mM AAPH for 2 h. EA.hy 926 cells were exposed to 20 mM AAPH for 2 h. Cells kept in complete medium without AAPH served as solvent control. Nrf2 translocation was evaluated for 2 h after treatment. Cells were stained with anti-Nrf2 antibody (green), nuclei are counterstained with Hoechst 33342 (blue). Images were analyzed using a Zeiss LSM 510 META. 2 h after exposure almost every endothelial cells shows strong colocalization of Nrf2 with the nucleus (red arrows). In control EA.hy 926 cells, no nuclear translocation was observed (compare white arrows). (PDF 13756 kb) [file 12989_2017_186_MOESM1_ESM.pdf]
