# Supplementary material for: Endothelial responses of the alveolar barrier in vitro in a dose-controlled exposure to diesel exhaust particulate matter
Source: Part Fibre Toxicol. 2017 Mar 6;14:7. doi: 10.1186/s12989-017-0186-4 (PMC5339948; doi:10.1186/s12989-017-0186-4)
Supplement: Additional file 2: Table S1. — Differential gene expression observed in EA.hy 926 cells exposed for 6 and 24 h to AAPH and tBHQ at different concentrations. The response factors are ratios between the gene expression levels (calculated relatively to the housekeeping genes) after exposure related to their respective solvent control (mean ± SEM, n = 3). Bold letters indicate significant differences between the evaluated time-points (P < 0.05 with a fold change greater than 2). (PDF 15 kb) [file 12989_2017_186_MOESM2_ESM.pdf]

	HMOX1		*HMOX2*		*SOD1*		*NQO1*		*HSP70*		*NRF2*	
	6 h	24 h	6 h	24 h	6 h	24 h	6 h	24 h	6 h	24 h	6h	24 h
AAPH 4mM	1.4 ± 0.07	**6.6 ± 0.3**	0.98 ± 0.06	0.9 ± 0.05	1.2 ± 0.3	1.4 ± 0.04	1.3 ± 0.07	1.7 ± 0.1	0.8 ± 0.05	0.02 ± 0.02	1.8 ± 0.08	1.1 ± 0.1
AAPH 20mM	1.6 ± 0.09	**25.7 ± 1**	0.95 ± 0.05	1 ± 0.03	1.1 ± 0.07	1.2 ± 0.1	1.3 ± 0.1	**2.2 ± 0.2**	0.5 ± 0.02	0.1 ± 0.07	1.8 ± 0.2	1.4 ± 0.1
AAPH 100mM	0.8 ± 0.06	NA	1.3 ± 0.1	NA	1.3 ± 0.1	NA	1.2 ± 0.1	NA	1.5 ± 0.1	NA	1 ± 0.1	NA
tBHQ 10 µM	1.4 ± 0.07	1.7 ± 0.1	1 ± 0.03	1.3 ± 0.1	0.9 ± 0.04	1 ± 0.06	1.8 ± 0.1	1.8 ± 0.1	1 ± 0.05	1 ± 0.08	0.9 ± 0.06	1 ± 0.07
tBHQ 40 µM	**2.9 ± 0.1**	**3.4 ± 0.4**	1 ± 0.05	1.5 ± 0.2	0.8 ± 0.04	0.9 ± 0.1	1.8 ± 0.1	**2.9 ± 0.4**	1.1 ± 0.06	1.4 ± 0.1	0.7 ± 0.05	0.9 ± 0.1
tBHQ 80 µM	**5 ± 0.3**	**6 ± 0.5**	1.2 ± 0.05	1.6 ± 0.1	0.8 ± 0.06	1.25 ± 0.08	**2.3 ± 0.1**	**2.8 ± 0.2**	1.6 ± 0.1	1.6 ± 0.1	0.9 ± 0.1	1.1 ± 0.1
