# Supplementary material for: Endothelial responses of the alveolar barrier in vitro in a dose-controlled exposure to diesel exhaust particulate matter
Source: Part Fibre Toxicol. 2017 Mar 6;14:7. doi: 10.1186/s12989-017-0186-4 (PMC5339948; doi:10.1186/s12989-017-0186-4)
Supplement: Additional file 3: Table S2. — Secretion of second messengers after exposure of the tetraculture to different doses of DEPM at different time-points. Tetracultures were exposed to 80 ng/cm2 or 240 ng/cm2 of diesel exhaust particles. Aliquots of the undernatant were taken at 6, 24 and 48 h after exposure and stored upon analysis at -80 °C. The level of secreted second messengers was evaluated using a multiplexed assay from Mesoscale Diagnostics for pro-inflammatory cytokines (A), cytokines (B) and chemokines (C). Tetracultures that were kept in the aerosol chamber for the same exposure time but with the particle generator in stand-by mode served as control. Data represents the mean of at least four Transwell™ inserts ± SEM. (PDF 125 kb) [file 12989_2017_186_MOESM3_ESM.pdf]

A **pro-inflammatory cytokines panel**

Cytokine		80 ng/cm^2			240 ng/cm^2		
		**6h**	**24h**	**48h**	**6h**	**24h**	**48h**
IFN-γ	*control*	7,5 ± 2,6	8,2 ± 2,4	8,8 ± 3,4	4,2 ± 0,9	8,1 ± 2,5	9,0 ± 3,9
	exposed	4,9 ± 2,7	8,5 ± 3,4	14,0 ± 3,7	2,9 ± 1,1	5,6 ± 1,7	10,1 ± 3,7
IL-10	*control*	3,9 ± 1,4	10,0 ± 5,7	6,3 ± 2,9	2,7 ± 1,0	7,0 ± 2,3	7,7 ± 4,0
	exposed	4,5 ± 1,3	5,3 ± 2,0	8,4 ± 2,8	3,2 ± 0,5	3,9 ± 1,4	6,6 ± 2,6
IL-12p70	*control*	3,2 ± 0,8	3,5 ± 0,9	5,0 ± 1,1	1,2 ± 0,1	3,3 ± 1,0	3,9 ± 1,4
	exposed	1,8 ± 1,0	3,4 ± 1,3	3,3 ± 1,4	1,1 ± 0,4	2,7 ± 0,9	3,7 ± 1,2
IL-13	*control*	48,4 ± 17,4	75,6 ± 17,3	38,6 ± 14,0	44,4 ± 9,5	65,3 ± 18,1	50,9 ± 20,8
	exposed	45,2 ± 9,4	43,6 ± 17,4	72,7 ± 23,4	43,6 ± 10,7	35,8 ± 11,1	51,3 ± 17,4
IL-1β	*control*	16,2 ± 2,9	31,2 ± 7,9	25,3 ± 4,9	28,1 ± 17,3	23,8 ± 5,7	16,1 ± 9,0
	exposed	9,8 ± 3,3	18,3 ± 7,1	16,1 ± 9,2	10,4 ± 2,4	13,7 ± 2,9	21,2 ± 4,6
IL-2	*control*	4,6 ± 3,3	6,6 ± 7,9	5,5 ± 4,9	2,8 ± 2,4	10,8 ± 5,7	25,5 ± 9,0
	exposed	14,2 ± 10,3	18,2 ± 13,7	31,2 ± 24,4	2,3 ± 0,6	12,0 ± 7,7	6,6 ± 2,2
IL-4	*control*	0,7 ± 0,2	1,2 ± 0,3	1,5 ± 0,3	0,3 ± 0,1	1,0 ± 0,4	1,0 ± 0,5
	exposed	0,5 ± 0,5	1,0 ± 0,4	0,9 ± 0,5	0,3 ± 0,2	0,8 ± 0,2	0,9 ± 0,4
IL-6	*control*	19,5 ± 5,7	34,3 ± 6,3	29,8 ± 7,5	19,4 ± 3,7	30,9 ± 10,1	22,7 ± 5,7
	exposed	27,9 ± 5,2	30,3 ± 4,5	39,6 ± 14,3	19,2 ± 6,0	25,5 ± 3,4	29,9 ± 6,7
IL-8	*control*	5815,4 ± 610,4	6183,4 ± 530,5	6236,1 ± 664,7	4718,9 ± 483,6	6163,3 ± 540,2	5530,7 ± 692,7
	exposed	4905,1 ± 753,8	5218,3 ± 681,0	4908,2 ± 672,8	4866,5 ± 806,9	5092,0 ± 596,0	4905,1 ± 784,7
TNF-α	*control*	4,0 ± 1,7	3,9 ± 0,4	10,3 ± 2,9	2,9 ± 0,7	4,6 ± 0,8	11,7 ± 2,8
	exposed	4,0 ± 1,4	6,2 ± 1,1	6,1 ± 0,9	3,1 ± 1,3	6,4 ± 1,6	8,0 ± 1,7

B

Cytokines panel

**Cytokine**		**80 ng/cm^2**			**240 ng/cm^2**		
		**6h**	**24h**	**48h**	**6h**	**24h**	**48h**
GM-CSF	*control*	10,9 ± 2,7	23,7 ± 1,4	81,0 ± 12,8	6,8 ± 1,5	23,9 ± 2,5	82,4 ± 5,2
	exposed	6,3 ± 1,5	24,6 ± 4,0	78,2 ± 12,6	23,0 ± 16,6	23,4 ± 2,5	83,7 ± 10,4
IL-12p40	*control*	1,3 ± 0,8	2,3 ± 0,7	3,6 ± 1,9	1,0 ± 0,3	2,0 ± 0,3	2,6 ± 0,8
	exposed	1,2 ± 0,3	2,0 ± 1,0	3,0 ± 0,6	0,8 ± 0,2	1,1 ± 0,3	1,9 ± 0,5
IL-15	*control*	1,3 ± 0,5	3,3 ± 0,4	7,0 ± 1,0	0,8 ± 0,1	3,1 ± 0,4	5,8 ± 1,0
	exposed	0,7 ± 0,2	3,0 ± 0,8	5,8 ± 1,4	1,6 ± 0,8	2,7 ± 0,4	5,2 ± 1,0
IL-16	*control*	5,5 ± 1,3	22,6 ± 4,2	28,4 ± 6,6	8,8 ± 3,8	17,3 ± 3,1	27,9 ± 7,8
	exposed	9,2 ± 1,9	17,6 ± 5,1	38,2 ± 6,1	6,0 ± 1,3	15,5 ± 3,5	30,4 ± 4,3
IL-17	*control* / *exposed*	**Not detectable under assay conditions**			**Not detectable under assay conditions**		
IL-1α	*control*	0,9 ± 0,2	7,0 ± 4,1	7,1 ± 2,3	19,9 ± 19,0	2,2 ± 0,3	8,5 ± 2,8
	exposed	1,5 ± 0,2	2,7 ± 0,6	6,6 ± 2,1	1,0 ± 0,2	3,0 ± 0,4	7,4 ± 2,0
IL-5	*control* / *exposed*	**Not detectable under assay conditions**			**Not detectable under assay conditions**		
IL-7	*control*	0,3 ± 0,1	1,4 ± 0,2	2,0 ± 0,4	0,6 ± 0,3	1,3 ± 0,2	2,3 ± 0,7
	exposed	0,6 ± 0,2	1,0 ± 0,3	2,7 ± 0,6	0,3 ± 0,1	1,0 ± 0,3	2,2 ± 0,5
TNF-β	*control*	0,0 ± 0,0	0,0 ± 0,0	0,0 ± 0,0	0,0 ± 0,0	0,1 ± 0,0	0,0 ± 0,0
	exposed	0,1 ± 0,0	0,1 ± 0,0	0,0 ± 0,0	0,1 ± 0,0	0,1 ± 0,0	0,0 ± 0,0
VEGF	*control*	29,3 ± 4,5	125,5 ± 17,2	357,3 ± 38,9	113,4 ± 76,2	110,1 ± 21,5	376,0 ± 52,2
	exposed	51,7 ± 18,2	114,2 ± 18,3	329,7 ± 55,0	39,1 ± 5,5	107,3 ± 12,4	330,2 ± 44,3

C

Chemokines panel

Cytokine		80 ng/cm² 6h			80 ng/cm² 24h			80 ng/cm² 48h			240 ng/cm² 6h			240 ng/cm² 24h			240 ng/cm² 48h		
Eotaxin	*control*	41,1	±	18,6	13,7	±	5,2	35,5	±	19,6	41,1	±	18,6	23,9	±	3,9	35,2	±	16,4
	exposed	20,9	±	6,9	24,1	±	8,2	30,5	±	8,1	13,4	±	4,6	19,1	±	5,6	78,3	±	26,4
Eotaxin3	*control*	6,4	±	2,6	9,8	±	3,3	11,5	±	4,0	4,6	±	0,7	5,8	±	0,8	18,1	±	6,0
	exposed	9,5	±	3,2	7,0	±	4,0	9,4	±	1,3	9,5	±	3,2	7,8	±	1,8	17,1	±	4,9
IP-10	*control*	16,4	±	6,9	46,9	±	14,7	170,2	±	29,1	16,4	±	5,4	44,2	±	8,4	165,3	±	16,8
	exposed	16,0	±	4,0	57,0	±	18,6	214,7	±	76,4	16,0	±	4,0	44,0	±	11,1	164,5	±	11,7
MCP-1	*control*	4832,0	±	1181,5	6000,2	±	1189,1	10545,1	±	1307,9	4832,0	±	1181,5	7428,2	±	1087,1	8985,3	±	942,1
	exposed	4696,7	±	1186,7	7813,6	±	492,7	8708,3	±	1186,8	4314,5	±	620,7	6973,6	±	472,9	8391,9	±	1128,5
MCP-4	*control*	14,5	±	6,0	14,0	±	2,5	19,4	±	2,9	12,8	±	4,4	20,2	±	3,6	20,1	±	3,4
	exposed	12,7	±	2,7	11,8	±	1,5	24,8	±	5,5	12,7	±	2,7	16,8	±	3,4	22,7	±	2,3
MDC	*control*	192,2	±	53,3	269,8	±	64,0	452,2	±	98,0	192,2	±	53,3	280,1	±	65,1	461,2	±	69,1
	exposed	218,6	±	97,3	284,2	±	76,9	439,8	±	108,4	185,8	±	61,6	270,1	±	59,6	415,2	±	50,1
MIP1a	*control*	201,6	±	134,2	83,3	±	29,8	56,5	±	18,7	134,9	±	66,3	115,8	±	47,0	61,6	±	18,2
	exposed	260,7	±	154,1	103,3	±	44,7	62,6	±	21,9	260,7	±	154,1	130,6	±	62,1	45,1	±	7,6
MIP1b	*control*	158,4	±	42,5	129,4	±	17,5	87,6	±	35,4	158,4	±	42,5	129,6	±	19,5	117,9	±	17,1
	exposed	184,3	±	63,3	142,8	±	29,6	82,4	±	18,7	118,7	±	39,2	216,3	±	35,5	75,7	±	23,4
TARC	*control*	70,2	±	46,3	147,1	±	48,0	82,2	±	32,1	51,2	±	24,5	116,5	±	48,9	80,6	±	50,5
	exposed	62,9	±	29,6	108,2	±	71,6	102,2	±	46,9	62,9	±	29,6	49,1	±	17,4	86,7	±	32,4
